# Nanotechnology for Biomedical Applications: Synthesis and Properties of Ti-Based Nanocomposites

**DOI:** 10.3390/nano15181417

**Published:** 2025-09-15

**Authors:** Maciej Tulinski, Mieczyslawa U. Jurczyk, Katarzyna Arkusz, Marek Nowak, Mieczyslaw Jurczyk

**Affiliations:** 1 Institute of Materials Science and Engineering, Faculty of Materials Engineering and Technical Physics, Poznań University of Technology, Jana Pawla II 24, 61-138 Poznań, Poland; maciej.tulinski@put.poznan.pl (M.T.); marek.nowak@put.poznan.pl (M.N.); 2 Division of Mother’s and Child’s Health, Poznań University of Medical Sciences, Polna 33, 60-535 Poznań, Poland; mujur@ump.edu.pl; 3 Department of Biomedical Engineering, Faculty of Engineering and Technical Sciences, University of Zielona Góra, Licealna 9, 65-417 Zielona Góra, Poland; k.arkusz@iimb.uz.zgora.pl

**Keywords:** nanotechnology, Ti-based nanocomposites, bioceramics, synthesis, consolidating techniques, scaffolds, surface modification

## Abstract

Nanobiocomposites are a class of biomaterials that include at least one phase with constituents in the nanometer range. Nanobiocomposites, a new class of materials formed by combining natural and inorganic materials (metals, ceramics, polymers, and graphene) at the nanoscale dimension, are expected to revolutionize tissue engineering and bone implant applications because of their enhanced corrosion resistance, mechanical properties, biocompatibility, and antimicrobial activity. Titanium-based nanocomposites are gaining attention in biomedical applications due to their exceptional biocompatibility, corrosion resistance, and mechanical properties. These composites typically consist of a titanium or titanium alloy matrix that is embedded with nanoscale bioactive phases, such as hydroxyapatite, bioactive glass, polymers, or carbon-based nanomaterials. Common methods for synthesizing Ti-based nanobiocomposites and their parts, including bottom-up and top-down approaches, are presented and discussed. The synthesis conditions and appropriate functionalization influence the final properties of nanobiomaterials. By modifying the surface roughness at the nanoscale level, composite implants can be enhanced to improve tissue integration, leading to increased cell adhesion and protein adsorption. The objective of this review is to illustrate the most recent research on the synthesis and properties of Ti-based biocomposites and their scaffolds.

## 1. Introduction

Medicine (including orthopedics) requires better solutions due to the aging society. Today, approximately 830 million people worldwide are aged 65 or above. According to the latest UN data, this population is projected to reach 1.7 billion by the year 2054 [1]. Aging societies and frequent injuries to the human body necessitate the search for biomaterials that can replace body parts and assume their functions (see Table 1) [2].

The unprecedented progress of technology forces the search for new biomaterials for use in new-generation devices, including medical ones. This research primarily focuses on biomaterials in the form of metallic, ceramic, polymer, and composite materials, mainly due to the relative ease of their preparation and the possibility of obtaining any desired shape. In addition, by selecting the chemical composition of biomaterials, the method, and the conditions of the production process, we can regulate their properties in a relatively wide range. Nanomaterials are the most desirable because they provide an optimum combination of mechanical, electrical, thermal, optical, chemical, and biological properties [3,4,5,6,7,8,9,10].

**Table 1:** Mechanical properties of some biomaterials and their densities [11,12,13,14,15,16].

Biomaterial (wt. %)	Tensile Strength (MPa)	Yield Strength (MPa)	Elongation (%)	Elastic Modulus (GPa)	Density (g/cm^3^)
Natural bone	35–283	–	1.07–2.10	5–23	1.85
α-type cp-Ti ann.	240	170	24	102.7	4.5
α + β-type Ti6Al4V ELI ann.	860–965	795–875	10–15	101–110	4.42
β-type Ti-15Mo ann.	874	544	21	78	4.95
Pure Mg	100.47	29.88	7.43	1.86	1.7
Mg-5Zn	194.59	75.60	8.50	36.47	1.735
316L stainless steel	580–1300	220–1200	50–60	190	7.9
Co-Cr-Mo alloy	650–1900	450–1600	5–20	210–253	8.3
Hydrogel sodium alginate PYG/A70	–	–	–	4.93	1.0
PMMA	8.16	–	1.04	7.85	1.17–1.20
PMMA/0.3 MWCNTs hybrid composite	30.99	–	4.72	6.57	–

Nanomaterials have gained significant popularity in medical applications over the past few years. Inorganic nanoparticles encompass a diverse range of materials, including metal oxides, elemental metals, their alloys, metal salts, fullerenes, carbon nanotubes, graphene, and their derivatives. Nanomaterials can exhibit enhanced corrosion, electrochemical and mechanical properties, biocompatibility, and bioactivity compared with their conventional microcrystalline counterparts. Many new nanostructured biomaterials have been developed and successfully tested in vivo and in clinical trials [9,10,17,18,19]. The market is currently limited in terms of available products. However, improvements in both short-term and long-term tissue integration of implants can be achieved by modifying the surface roughness at the nanoscale level to enhance protein adsorption and cell adhesion. Additionally, biomimetic calcium phosphate coatings can be used to enhance osteoconduction, while the addition of biological drugs can accelerate the bone healing process in the peri-implant area.

The development of advanced biomaterials is critical to the continued progress of biomedical implants and devices, particularly in orthopedic and cardiovascular applications. Novel materials are constantly being engineered to strike a balance between mechanical performance, biocompatibility, and cost-effectiveness—three essential parameters governing their clinical viability. Among these emerging materials, titanium-based nanocomposites, magnesium-based materials, Mg-Ti alloys and hybrids, as well as polymer-based hybrids, have shown significant promise. Each class offers unique advantages: Ti-based nanocomposites are known for their exceptional mechanical strength and corrosion resistance; Mg-based materials offer biodegradability and elastic modulus similar to natural bone; Mg-Ti hybrids aim to combine the strengths of both components; and polymer-based hybrids provide high versatility and cost-effectiveness, often with tailored biodegradability.

Table 2 provides a comparative summary of these materials across three critical domains—mechanical strength, biocompatibility, and cost—to aid researchers, engineers, and clinicians in selecting the most appropriate material for specific biomedical applications.

Nanobiocomposites, as a new class of materials at the nanoscale dimension, are foreseen to change tissue engineering and bone implant applications due to their improved corrosion resistance, mechanical properties, biocompatibility, and antimicrobial activity [25,26,27,28,29,30,31,32,33]. Nanotechnology has gained popularity in various fields beyond implantology, including soft and hard tissue reconstruction, tooth regeneration, bone repair, periodontal therapy, plaque control, caries diagnostics, and caries treatments [5,6,10,26,34].

Great attention has also been paid to the surface treatment applied to metallic biomaterials, which provides faster osseointegration and improves mechanical properties [31,32,35]. This paper reviews the synthesis and properties of Ti-based nanocomposites and their scaffolds.

## 2. Classification of Synthesis Methods

**Figure 1:**
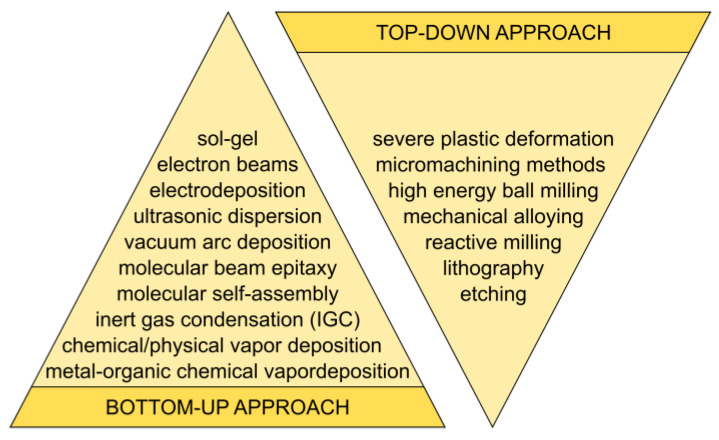
Bottom-up and top-down methods.

**Figure 2:**
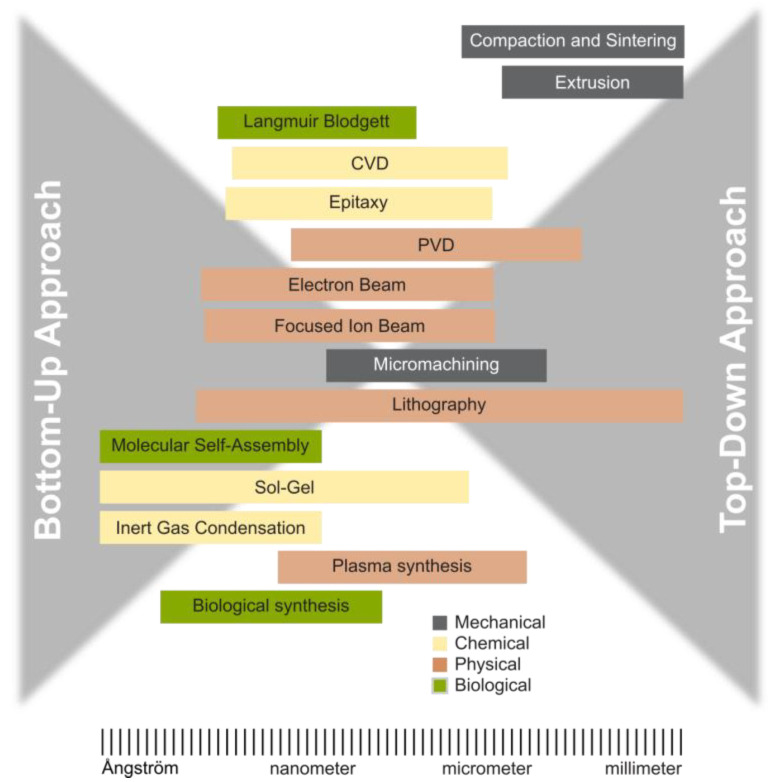
Schematic illustration depicting the formation of nanostructures.

There are two primary methods for producing nanoparticles, as depicted in Figure 1 and Figure 2 [36,37,38,39,40]. The conventional method of producing fine particles, referred to as the ‘top-down’ approach, entails reducing particle size through attrition and various comminution techniques. Conversely, the ‘bottom-up’ approach, which has garnered increasing prominence in recent years [41], entails constructing a material layer sequentially, atom by atom, molecule by molecule, or cluster by cluster.

Both approaches play a crucial role in modern industry, and they are likely to be instrumental in bionanotechnology as well [40]. However, they have their advantages and disadvantages. The top-down approach encounters challenges, including imperfections in the surface structure and substantial crystallographic damage to the processed patterns. Despite these defects, they continue to be important in synthesizing nanostructures.

The bottom-up approach, which is widely favored in nanotechnology, is not a new concept. It is currently widely used in the fabrication and processing of nanostructures. It is crucial to remember that the characteristics of the materials produced can vary greatly depending on the method employed in their fabrication. Apart from direct atom manipulation, numerous well-established methods exist for producing nanomaterials, including mechanical, chemical, physical, and biological processes (Table 3) [42,43].

Additionally, there are distinct definitions of manufacturing and the synthesis of nanomaterials. Synthesis refers to the creation of novel nanomaterials with unique properties. For example, a synthesis endeavor employs robust physics to identify and target specific materials, shapes, and heterostructures that will yield desirable or unique properties [36].

In contrast, manufacturing entails the production of products featuring nanostructures constructed at the molecular level of matter. This process is referred to as nanomanufacturing. The primary objective of nanomanufacturing is to scale up production, guarantee reliability, and minimize costs in the manufacturing of nanoscale materials. It also encompasses research, development, and the integration of top-down processes and progressively intricate bottom-up or self-assembly processes. In essence, nanomanufacturing results in the production of enhanced materials and novel products.

Recently, several research groups have investigated the utilization of biological systems for the synthesis of nanoparticles (Table 4) [44,45,46].

Biological methods for nanoparticle synthesis offer a significant advantage by circumventing the stringent processing conditions typically associated with physical and chemical methods. These methods frequently require extreme temperatures, pressures, and pH levels, resulting in substantial costs. In contrast, biological approaches facilitate synthesis at physiological conditions, thereby reducing costs and ensuring the safety of the nanoparticles. The rapid growth of biological synthesis of nanoparticles with controlled toxicity is an eco-friendly approach.

## 3. Nanomaterial Synthesis Methods

### 3.1. Physical Methods

Physical methods for producing nanomaterials utilize mechanical, thermal, or electromagnetic processes that circumvent chemical reactions. These techniques are typically categorized as top-down approaches, wherein bulk materials are fragmented into nanoscale particles. They are not limited to top-down approaches, as certain physical methods facilitate controlled growth of nanostructures under specific physical conditions. A few examples are given below.

#### 3.1.1. Ion Beam Techniques

Ion beam techniques employ advanced physical methods to produce or modify nanomaterials by bombarding materials with high-energy ion beams (typically within the keV to MeV energy range). These techniques enable precise control over surface structure, composition, and nanoscale patterning, making them indispensable in nanotechnology and materials science in general [47].

**Figure 3:**
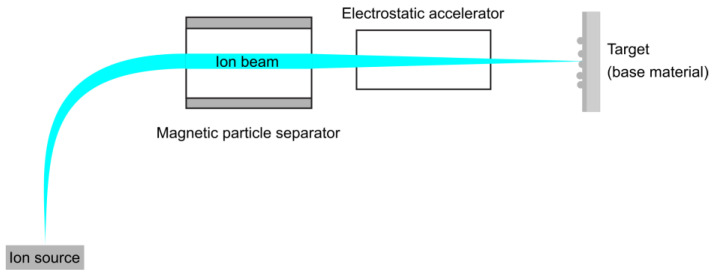
Schematic of an ion implantation system.

Ion implantation involves injecting very energetic ions into the surface of a solid substrate. The main parts of an ion implantation facility, as depicted in Figure 3, include an ion source, a vacuum system, a magnetic analyzer, an accelerator, a beam scanning system, and a target chamber.

To prevent ion scattering and contamination of the ion beam and target surface with unwanted ions, the complete ion implantation system must be equipped with ultra-high vacuum (UHV) technology. The ion source generates ions using a secondary energy source to ionize a working gas, such as nitrogen or methane.

The magnetic analyzer, a device that uses magnetic fields to separate ions, accelerates them through a flight tube. Based on their charge-to-mass ratios, ions are separated and then accelerated to high energies in an acceleration tube. This acceleration tube typically has an acceleration voltage ranging from 30 to 300 kV. After acceleration, the ions are electrostatically focused into a spatially defined beam that is then directed towards the surface of the target.

Ion implantation techniques offer a high quenching rate, enabling chemical and microstructural engineering at the atomic level. Their primary advantages include precision (ranging from atomic to nanoscale resolution) and flexibility. Additionally, these techniques can produce metastable crystalline and amorphous phases, non-equilibrium solid solutions, and nanocrystalline materials.

Ion beam implantation of various ions, followed by the formation of a hydroxyapatite (HA) coating on the titanium system, has been extensively studied [48]. The properties of these coatings have been thoroughly investigated. Besides calcium and phosphorus, other ions can also be implanted, leading to enhanced corrosion resistance, wear resistance, and improved implant–tissue interactions.

#### 3.1.2. Plasma Synthesis

Plasma synthesis of nanomaterials offers a versatile approach that utilizes plasma, a highly ionized gas comprising ions, electrons, and neutral particles, to synthesize nanoparticles or nanostructures. Its significance lies in its non-equilibrium chemistry, elevated temperatures, and reactive environment, which facilitate the synthesis of metals, metal oxides, nitrides, carbides, and even carbon-based nanomaterials such as graphene or nanotubes.

In the nano-synthesis process, materials are initially heated to the point of evaporation using induction plasma. Subsequently, the generated vapors are rapidly quenched within the quench/reaction zone. The gas employed for quenching can be inert gases such as Ar and N_2_ or reactive gases such as CH_4_ and NH_3_. The produced nanometric powders are typically collected using porous filters, which are strategically positioned away from the plasma chamber section. Due to the high reactivity of metal powders, meticulous attention is required to ensure their proper pacification before removing the collected powder from the filtration section of the process.

**Figure 4:**
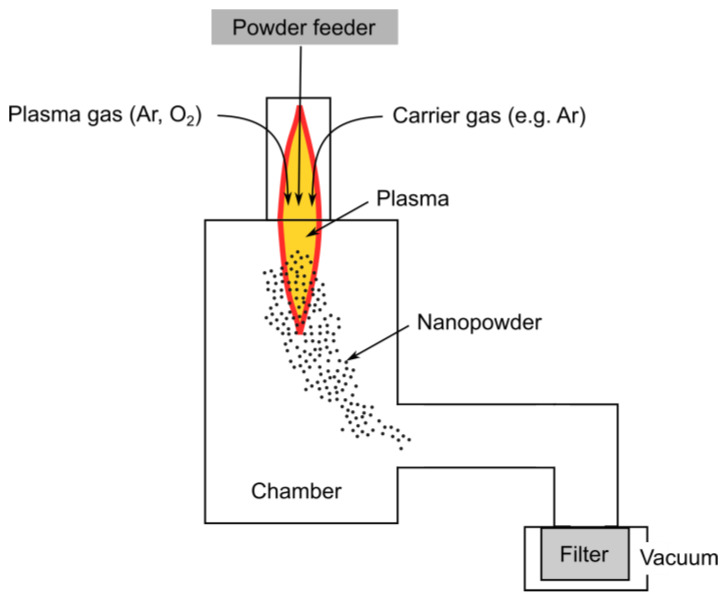
Schematic of a plasma synthesis system.

The induction plasma system has demonstrated its efficacy in the synthesis of nanopowders. The standard arrangement of the plasma process is illustrated in Figure 4. The size range of the produced nanoparticles spans from 20 to 100 nanometers, contingent upon the quench conditions employed. The efficiency exhibited by the system ranges from a few hundred grams per hour to approximately 3 to 4 kg per hour, commensurate with the distinct physical properties of the materials being synthesized.

To enhance the surface properties of metals and expand their applications, various methods have been employed to modify their surfaces. For instance, the properties of titanium can be modified through boride microplasma surface alloying [49]. This process offers a wide range of layer thicknesses, which can be controlled by the amount of deposited powder and process parameters, and it can be an effective method to produce a TiB phase dispersed in an α-Ti matrix with exceptional hardness and good cytocompatibility.

#### 3.1.3. Pulsed Laser Deposition

**Figure 5:**
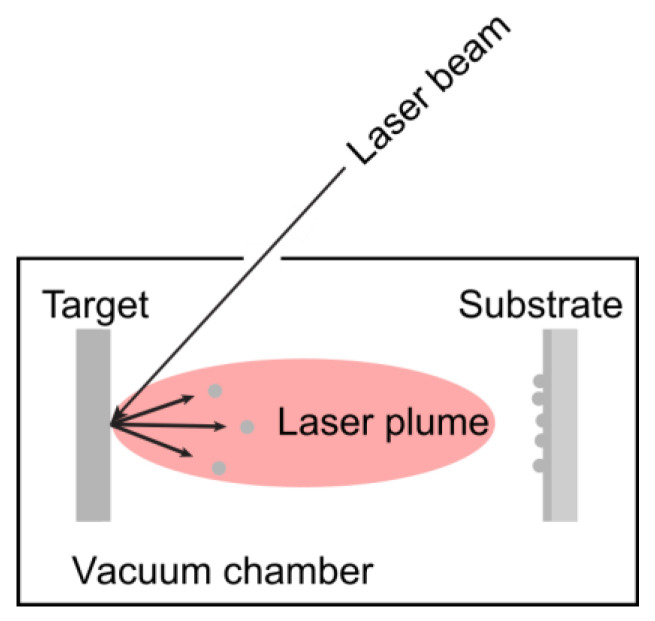
Schematic diagram of pulsed laser deposition.

Pulsed laser deposition (PLD) is a simple and versatile physical method for depositing thin films [50]. This technique, based on plasma vapor deposition (PVD) processes, involves the impact of short-pulsed, high-power laser radiation on solid targets. The target and the substrate facing it are held in the chamber, as depicted in Figure 5. The laser beam enters a vacuum chamber and strikes the target. The removal of atoms from the bulk material occurs through the vaporization of the bulk in the surface region in a non-equilibrium state. PLD enables the deposition of multilayer structures and allows for film growth in reactive environments containing various gases.

Advantages of this technique include excellent stoichiometric transfer from target to film, compatibility with complex materials (e.g., ferroelectrics), and flexibility in film thickness control (nm to micrometers) [51]. It also allows growth under various atmospheres (O_2_, Ar, N_2_). The primary limitation is the challenge of scaling up for large-area deposition.

The PLD technique has numerous applications, including the fabrication of nano-scale metal oxide thin films [52]. Notably, PLD has been shown to produce high-quality crystalline films that surpass those obtained using other thin film growth techniques.

#### 3.1.4. Physical Vapor Deposition

Physical vapor deposition is a vaporization coating technique that transfers material on an atomic level. It is similar to CVD, but the main difference is that the material to be deposited (raw materials or precursors) starts in solid form, unlike in CVD, where it is introduced to the reaction chamber in the gaseous state. Sputtering and pulsed laser deposition processes are also included in PVD.

PVD processes, conducted under vacuum conditions, follow four main steps: evaporation, transportation, reaction, and deposition. In the initial stage, a target material, containing the substance to be deposited, is bombarded with a high-energy source, such as a beam of electrons or ions. This bombardment “vaporizes” atoms from the target’s surface. Subsequently, these “vaporized” atoms are transported from the target to the substrate to be coated. This transportation typically occurs on straight trajectories.

Coatings can be made of carbides, nitrides, metal oxides, and other materials. In these cases, the target material is the metal, and its atoms react with the appropriate gas during the transportation stage. For instance, reactive gases like oxygen, nitrogen, and methane may be involved. PVD coatings are deposited for various reasons, such as enhancing hardness and wear resistance, reducing friction, and improving oxidation resistance.

There are several different PVD techniques, differing in the way the energy is delivered.

**Figure 6:**
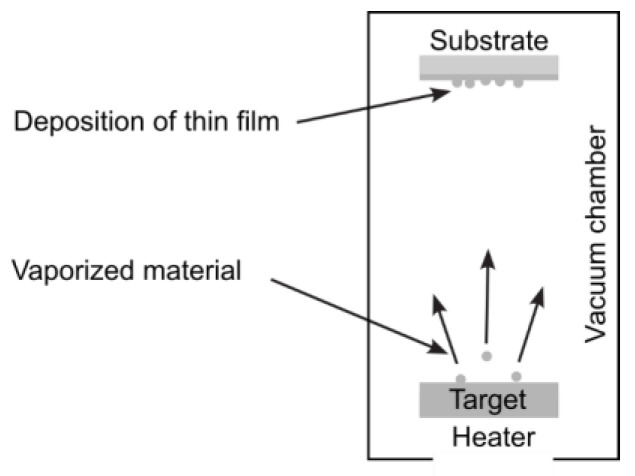
Schematic of thermal evaporation system.

Thermal evaporation, a practical application of the physical vapor deposition technique (Figure 6), involves heating a substance to a suitable temperature using energy sources such as electrical currents, lasers, electron beams, or arc dischargers. This temperature is typically as high as 100 °C to ensure well-defined film properties. The thermally released atoms or molecules then leave the surface of the evaporated material and adhere to the substrate or surrounding walls, forming a coating [53].

Another PVD technique, called the sputtering deposition process, involves the emission of neutral atoms, clusters, molecules, or ions from the material bombarded by energetic particles. This enables the sputtering of virtually any substance.

**Figure 7:**
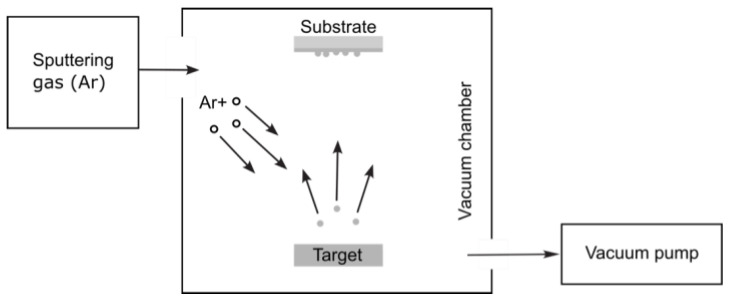
Schematic of the sputtering system.

The sputtering process is illustrated schematically in Figure 7. By ionizing the sputtering gas through a glow discharge, positive ions bombard the target surface, ejecting neutral atoms. These ejected atoms leave the solid by impulse transfer, resulting in relatively large energies (approximately 5–40 eV), unlike evaporation, where the evaporated atoms possess energies ranging from 0.2–0.3 eV. Subsequently, the ejected atoms condense on the substrate surface, forming a thin film [54].

**Figure 8:**
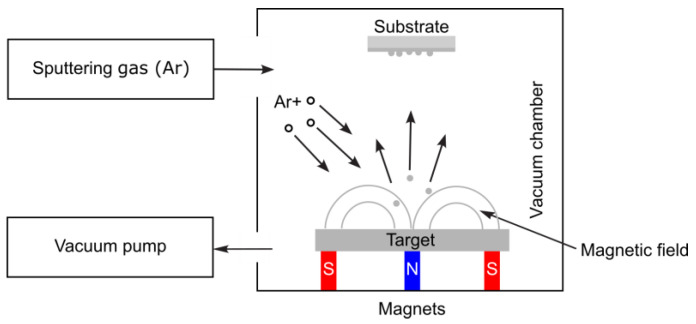
Schematic of a magnetron sputtering system.

Direct current sputtering (DCS) was initially used for sputtering conducting materials. Later, it evolved into various configurations, including radio-frequency sputtering, bias-sputtering, and a triode system. The most widely used sputtering system is the magnetron configuration. As depicted in Figure 8, a magnetic field confines the plasma around the target’s surface [55].

Magnetron sputtering is a well-established coating technique utilized mostly in microelectronic applications, particularly for metallization and magnetic device fabrication, but recently also in the field of nanobiomaterials. For instance, a ZnTiO_3_-ZnO nanocomposite coating was deposited on Ti_6_Al_4_V substrates by a single-step, low-temperature synthesis route using reactive magnetron co-sputtering of pure titanium and zinc targets. This helped create a more biocompatible material with antibacterial properties [56]. Magnetron sputtering techniques were also utilized to deposit Ti_x_N_y_ thin films on biological TC4 substrate in a mixed atmosphere of Ar and N_2_. It resulted in higher mechanical properties, bio-corrosion resistance, and bio-compatibility, and it can be implemented in orthopedic implant devices and neural electrodes [57].

### 3.2. Chemical Methods

Chemical methods for producing nanomaterials involve controlled chemical reactions to create particles with dimensions in the nanometer range. These methods include sol–gel, chemical vapor deposition, colloidal dispersion, epitaxial growth, hydrothermal route, microemulsions, polymer route, and other precipitation processes. These methods are widely used due to their scalability, cost-effectiveness, and ability to control particle shape, size, and composition. Below are some common chemical synthesis methods.

#### 3.2.1. Sol–Gel

The sol–gel method is employed to synthesize nanomaterials, particularly metal oxides, by transitioning a system from a liquid “sol” (a colloidal suspension of particles) to a solid “gel” phase [58,59].

**Figure 9:**
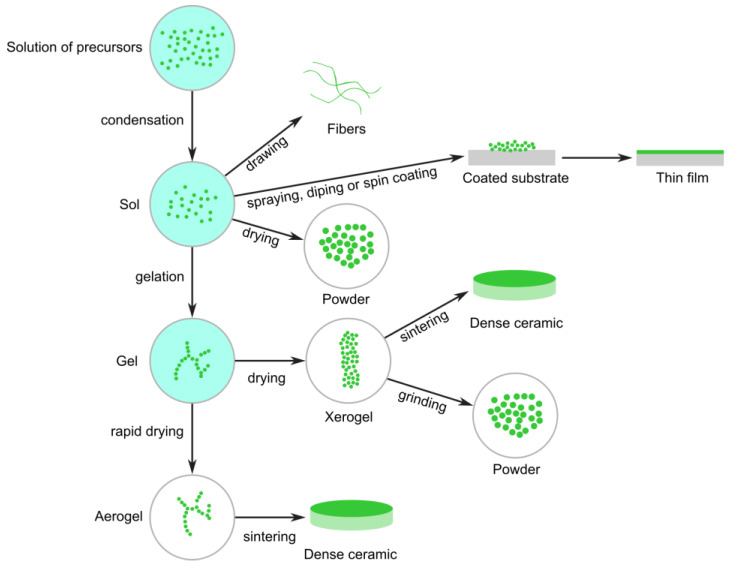
Diagram of the sol–gel method.

In this process, metal alkoxides or metal salts serve as precursors. These undergo hydrolysis and condensation reactions, leading to the formation of a network structure that traps the solvent. Subsequently, drying and calcination (heat treatment) transform the gel into a dense nanomaterial, often manifesting as nanoparticles, thin films, or porous materials. The removal of liquid from the sol results in the formation of a gel. This transition between sol and gel controls the particle size and shape. Calcination of the gel yields the oxide (Figure 9).

The sol–gel method enables the production of glasses at significantly lower temperatures compared to conventional melting. This process also facilitates the synthesis of compositions challenging to obtain through conventional methods due to issues related to high melting temperatures or crystallization. Moreover, the sol–gel approach is a high-purity process that results in exceptional homogeneity. The most successful applications leverage the method’s ability to control composition, microstructure, purity, and uniformity, coupled with its capacity to form various shapes at low temperatures. Furthermore, the sol–gel approach is adaptable to producing films, fibers, and bulk pieces (Figure 9). Additionally, sol–gel technology is utilized in other critical applications, including the creation of controlled porosity and high surface area for catalyst supports, porous membranes, and thermal insulation.

Sol–gel technology, renowned for its advantages of low processing costs, energy efficiency, high production rates, and rapid productivity of fine, homogeneous powders, has garnered widespread adoption. It serves as an effective process for producing nanoparticles, such as Ag-TiO_2_, Fe_2_O_3_, ZnO, and TiO_2_.

#### 3.2.2. Chemical Vapor Deposition

**Figure 10:**
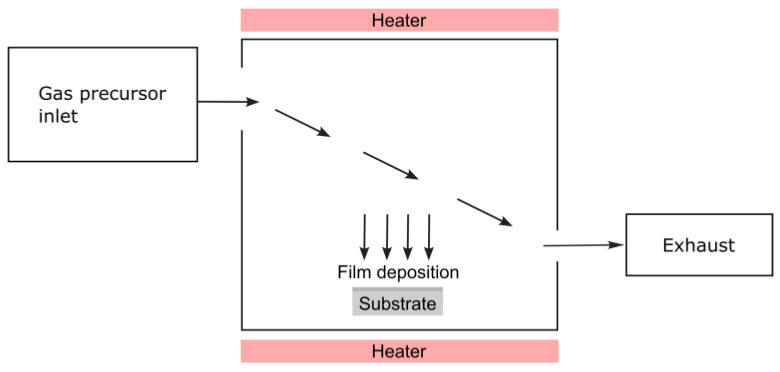
Diagram of a CVD system.

Chemical vapor deposition (CVD) is a materials processing technique in which a thin solid film is formed on a substrate through chemical reactions of gaseous precursors [60]. It can also be used to produce powders and high-purity bulk materials, as well as composite materials [61,62]. This method is highly valued for producing high-purity, high-performance solid materials, and it plays a crucial role in various industries, including semiconductors, photovoltaics, optics, and coatings. As depicted in Figure 10, a chamber contains a heated object (or objects) to be coated. Chemical reactions occur on and near the hot surfaces, leading to the deposition of a thin film on the surface. This process also produces chemical by-products that are exhausted from the chamber along with unreacted precursor gases. Given the diverse range of materials deposited and applications, CVD has numerous variants. These variants can be performed in hot-wall reactors or cold-wall reactors, with total pressures ranging from sub-Torr to above-atmospheric levels. Carrier gases can be used, and temperatures typically range from 200–1600 °C. Additionally, enhanced CVD processes can utilize ions, plasmas, lasers, photons, hot filaments, or combustion reactions that help increase deposition rates and/or lower deposition temperatures [63].

As an example, the CVD process provides deposited titanium layers. To improve the process, reaction gases are titanium tetrachloride and a hydrocarbon gas, which for a preferred embodiment of the process, is methane. The chemical reaction is as follows: TiCl_4_ + 4CH_4_ = Ti + 4CH_3_Cl + 2H_2_ [64].

Chemical vapor deposition offers several advantages as a method for depositing thin films. Firstly, CVD films are generally quite conformal, meaning they adhere closely to the substrate. Secondly, materials can be deposited with exceptionally high purity and relatively high deposition rates. However, the safety of the materials used in CVD is a significant disadvantage. Some precursors and by-products can be toxic, pyrophoric, or corrosive, posing potential health risks to the operator.

Nonetheless, CVD continues to be a cornerstone of modern materials science, enabling the precise fabrication of thin films and nanostructures essential for advanced technologies.

#### 3.2.3. Hydrothermal Route

The hydrothermal method, a solution-based synthesis technique, produces nanomaterials under high pressure and moderate-to-high temperatures in a sealed vessel called an autoclave. It is particularly effective for growing crystalline materials that are challenging to synthesize using conventional methods.

The shape, size, and crystallinity of metal oxide nanoparticles or nanostructures can be controlled and altered by adjusting specific parameters, such as reaction temperature, reaction time, solvent type, surfactant type, and precursor type [65].

**Figure 11:**
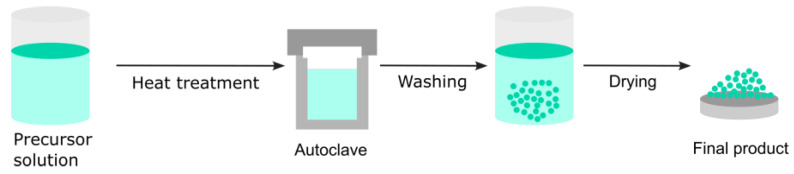
Diagram of a hydrothermal route method.

In the hydrothermal process, chemical reactions occur in a water-based solution at temperatures typically between 100 °C and 300 °C under autogenous pressure (Figure 11). The high temperature enhances the solubility and reactivity of materials, facilitating the nucleation and controlled growth of nanocrystals.

The hydrothermal route enables the processing of highly homogeneous nanoparticles and is one of the most appealing methods for processing nanohybrid and nanocomposite materials. It can be successfully utilized to synthesize nanostructured titanium dioxide, graphene, carbon, and other nanomaterials [66]. These materials find applications in a diverse range of technological fields, including biomedical and biophotonics.

#### 3.2.4. Epitaxial Growth

Epitaxy, a highly controlled method of depositing a crystalline layer of material on a crystalline substrate, is characterized by its ordered growth of single-crystalline material. The deposited layer adheres to the crystal structure and orientation of the underlying substrate, making it a widely used technique in nanotechnology and the semiconductor industries. Epitaxy enables the production of high-quality nanomaterials such as thin films, nanowires, and quantum dots with precise structural and electronic properties [67].

Some of the most common epitaxial techniques include liquid phase epitaxy (LPE), hydride vapor phase epitaxy (HVPE), metal organic vapor phase epitaxy (MOVPE), ultra-high vacuum chemical vapor deposition (UHV-CVD), chemical beam epitaxy (CBE), and molecular beam epitaxy (MBE) [68].

#### 3.2.5. Polymer Route

The polymer route is a versatile and widely used method for synthesizing nanomaterials using polymers as templates, precursors, stabilizers, or reaction media. This method allows for precise control over the size, shape, and dispersion of nanomaterials, making it particularly useful for producing metal, metal oxide, and composite nanoparticles.

**Figure 12:**
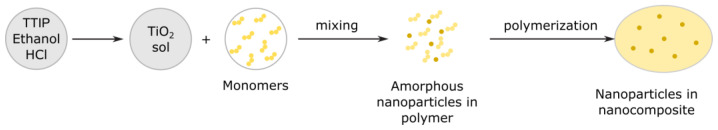
Schematic representation of the synthesis of nanoparticles in situ within a polymer matrix.

Nanocomposites can be prepared through two main methods: in situ synthesis of inorganic particles or dispersion of fillers in a polymeric matrix (Figure 12) [69]. The choice of preparation technique is crucial to obtaining nanomaterials with desired properties. The synthesis of polymer nanocomposites typically employs bottom-up or top-down methodologies.

Recently, a series of transition metal ion-doped TiO_2_ composites have been prepared via polymer-induced self-assembly [70]. For example, the synthesized Zr-doped TiO_2_ nanocomposite exhibits several notable characteristics, such as uniform distribution of pore sizes, high surface area, and relatively strong surface acidity.

#### 3.2.6. Colloidal Dispersion

The colloidal dispersion method is a widely used technique for synthesizing nanomaterials, particularly nanoparticles. It involves forming and stabilizing nanoparticles within a liquid medium, usually water or an organic solvent, to create a stable suspension of particles in the nanometer size range.

Colloidal dispersion is a heterogeneous system composed of a dispersed phase and a dispersion medium. In this system, one substance is dispersed as very fine particles in another substance called the dispersion medium [71].

Numerous methods and procedures can be applied to obtain a wide range of compounds containing nanoparticles for biological applications. For example, iron oxide nanoparticles, graphene oxide nanosheets, and TiO_2_ nanoparticles can be suspended.

#### 3.2.7. Microemulsions

**Figure 13:**
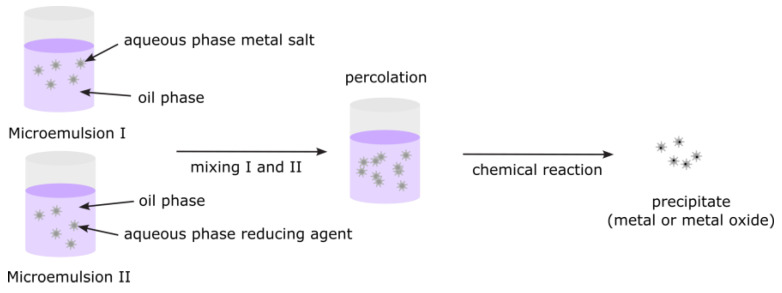
Microemulsion process.

The microemulsion method, a powerful and widely used wet chemical technique, is particularly effective for synthesizing nanomaterials, especially nanoparticles. Among the various synthesis processes, the microemulsion method stands out as a versatile technique that allows for precise control over particle properties such as size, geometry, homogeneity, morphology, and surface area (Figure 13) [72]. This technique exploits thermodynamically stable microemulsions—transparent, isotropic mixtures of oil, water, surfactants, and sometimes cosurfactants—to control the nucleation and growth of particles at the nanoscale. Microemulsions are isotropic, macroscopically homogeneous, and thermodynamically stable solutions containing at least three components: a polar phase (usually water), a nonpolar phase (usually oil), and a surfactant. On a microscopic level, surfactant molecules form an interfacial film that separates the polar and non-polar domains. This layer can form various microstructures, ranging from droplets of oil dispersed in a continuous water phase (O/W-microemulsion) to a bicontinuous “sponge” phase, and finally, water droplets dispersed in a continuous oil phase (W/O-microemulsion).

The microemulsion method has been instrumental in the synthesis of various colloidal metals, including AgCl, Fe_3_O_4_, TiO_2_, Fe_2_O_3_, and Al_2_O_3_.

#### 3.2.8. Solvothermal Decomposition

**Figure 14:**
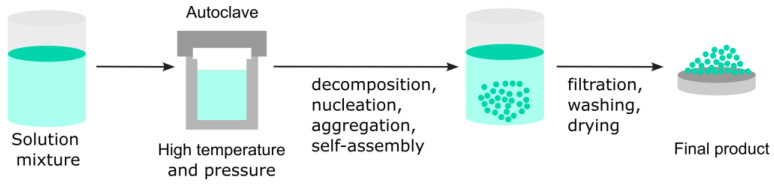
Schematic diagram of the solvothermal process.

Solvothermal decomposition, a widely used method in nanomaterial production, particularly for synthesizing metal oxides, sulfides, and other inorganic nanoparticles, combines solvothermal synthesis and thermal decomposition [73]. This technique employs a solvent under high-temperature and -pressure conditions to drive the decomposition of a precursor material into nanoparticles (see Figure 14).

The process begins by dissolving a metal-containing precursor in a suitable solvent. This mixture may also contain capping agents, surfactants, or complexing agents to control the nucleation and growth of nanoparticles. Precursors are typically metal acetates, chlorides, alkoxides, or organometallic compounds.

In the next step, the reaction mixture is sealed in a Teflon-lined stainless steel autoclave to allow the reaction to occur at temperatures above the boiling point of the solvent, creating high pressure (approximately 10 to 100 atm). As the temperature increases, the precursor decomposes thermally. The decomposed metal ions or molecular fragments then nucleate to form small clusters. These nuclei act as seeds for further growth. When the reaction is complete, the autoclave is cooled to room temperature, and the nanoparticles are recovered by centrifugation, filtration, or precipitation. They are often washed (with alcohol, water, etc.) to remove unreacted species or residual organics. Sometimes, they are annealed to improve crystallinity or remove surfactants.

Under solvothermal conditions, materials typically form highly crystalline structures. The solvent molecules, ligands, and reaction parameters can influence the morphology (shape) of the nanocrystals, creating spheres, rods, cubes, flowers, and so on [74].

Recently, high-quality crystalline 2D graphene-like anatase-TiO_2_ nanosheets were produced by an air-gap assisted solvothermal approach [75].

### 3.3. Mechanical Methods

Mechanical methods for producing nanostructures involve the creation of fine-grained powders, which can be as small as the nanometer scale [6,7,8,9,10,17,18,19,25,26,34,35,39,40,76,77,78,79,80,81,82]. These techniques offer several advantages, including their simplicity, low-cost equipment requirements, and the ability to process coarse feedstock powders. However, challenges such as powder agglomeration and contamination from equipment pose difficulties. Mechanical processes are typically employed for inorganic materials, metals, their alloys, and composites.

#### 3.3.1. Milling Processes

Milling has always had three main goals: mixing or blending, changing particle shape and size, and reducing particle size [6,7,10,76,77,79]. Ball mills combine crushing/shearing and impact forces in different proportions, depending on the type of ball mill used. Various types of ball mills have been developed for different purposes, such as tumbler mills, attrition mills, shaker mills, vibratory mills, and planetary mills.

The milling process that produces a nanometric structure occurs approximately 10 times faster in a shaker ball mill compared to a planetary ball mill. The time required to produce powder with a nanostructure depends not only on the material’s properties but also on other factors, such as the weight of the milling balls and the ratio of the weight of the balls to the weight of the powder (BPR). The ball-to-powder weight ratio can range from 1 to 100.

During the milling of materials in high-energy ball mills, three major processing methods can be employed: (i) high-energy ball milling (HEBM), (ii) mechanical alloying (MA), and (iii) mechanochemical synthesis (MCS) [7,39,76,77,79].

#### 3.3.2. High-Energy Ball Milling

High-energy ball milling is a top-down nanotechnology approach for synthesizing nanoparticles. In this process, brute force is applied to the material, which can be a metal, a pre-alloyed intermetallic, or a solid chemical (stoichiometric) compound. By breaking thin chemically passive surface coatings, such as surface oxides, fresh, clean, chemically active metallic surfaces can be exposed. However, this milling can also introduce defects into solid compounds. HEBM, except for decreasing particle size, produces microdeformation of the crystal lattice of the ground material. The finest grinding can be achieved using a liquid milling medium.

#### 3.3.3. Mechanical Alloying

**Figure 15:**
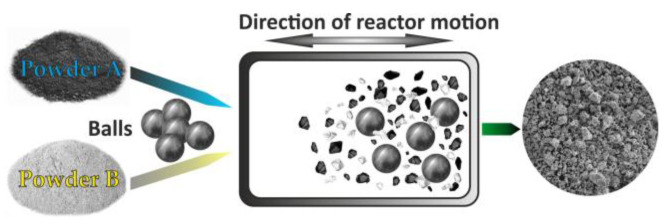
Cross-sectional schematics of the MA (shaker mixer mill).

Mechanical alloying is a process that involves milling a mixture of elemental metal powders or powders of metal and nonmetal for an extended period. This process triggers the solid-state alloying of the elemental powders [76,77,78,79]. The MA process has been successfully employed to prepare a diverse range of alloy powders, including those exhibiting supersaturated solid solutions, quasicrystals, amorphous phases, and nano-intermetallic compounds [78,79]. This technique has been demonstrated as a novel approach for alloy formation (Figure 15) [7,27,28,29,30,31,32,33,83,84,85].

**Figure 16:**
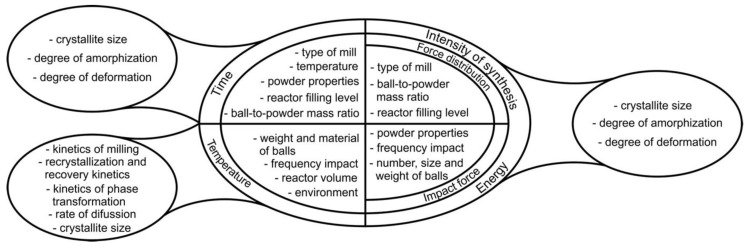
The parameters of the MA process.

The starting materials utilized in the MA are commercially available as high-purity powders, exhibiting sizes within the range of 1 to 100 μm. The sequence of simultaneous mechanical and chemical events can be expressed as follows [7,76,85]: mechanical stressing, severe plastic deformation, formation of a submicron lamellar microstructure, cold welding, fracture, formation of nanostructure, extended solid solubility, mechanical alloying with formation of thermodynamically stable and/or metastable phases, and amorphization. In Figure 16, the correlation between the different parameters of the MA process is presented.

Furthermore, surface and interface contamination is a major concern for all nanocrystalline materials prepared through various synthesis routes. During mechanical attrition, contamination by the milling tools (mostly iron) and the atmosphere (trace elements of oxygen and other gases) can be particularly problematic [86,87]. By minimizing the milling time and using the purest, most ductile metal powders available, a thin coating of the milling tools can be obtained using the respective powder material. This coating significantly reduces Fe contamination. After loading the powder in an inert gas glove box, the vial can be sealed with an O-ring to minimize or eliminate atmospheric contamination.

Using X-ray photoelectron spectroscopy (XPS) and Auger electron spectroscopy (AES), the cleanliness of the surface of nanocrystalline alloys prepared using MA method was examined [86,87]. The iron and oxygen contents in mechanically synthesized nanomaterials were less than 1–2% and 300 ppm, respectively.

#### 3.3.4. Mechanochemical Synthesis

Mechanochemical synthesis can produce both stable and metastable phases in two-component and multicomponent systems [88]. This process involves a strong mechanical force used to material destruction and leads to the formation of a distinct structure. A typical machine employed in the current application of the mechanochemical process is a planetary ball mill. During this process, the particle size diminishes, and surface energy increases. The most appealing characteristic of the mechanochemical method is its ease in synthesizing nanostructured materials.

### 3.4. Severe Plastic Deformation

Top-down approaches involve the fragmentation of coarse grains to ultra-fine grains (UFG) using severe plastic deformation techniques (SPD) [89,90]. Currently, there is a growing demand for the development of UFG metallic materials for biomedical applications due to their improved properties compared to their bulk alloys.

Severe plastic deformation techniques, such as equal channel angular pressing (ECAP) [89], cyclic extrusion compression (CEC) [91], high-pressure torsion (HPT) [92], twist extrusion (TE) [93], friction stir processing (FSP) [94], and multi-directional forging (MDF), also known as multiaxial compression/forging (MAC/F) [95] with the potential for intensive straining, are used to obtain nanocrystalline materials. In addition, there are several methods for producing ultra-fine grain sheets, such as accumulative roll bonding (ARB) [96] and repeated corrugation and straightening (RCS) [97].

**Figure 17:**
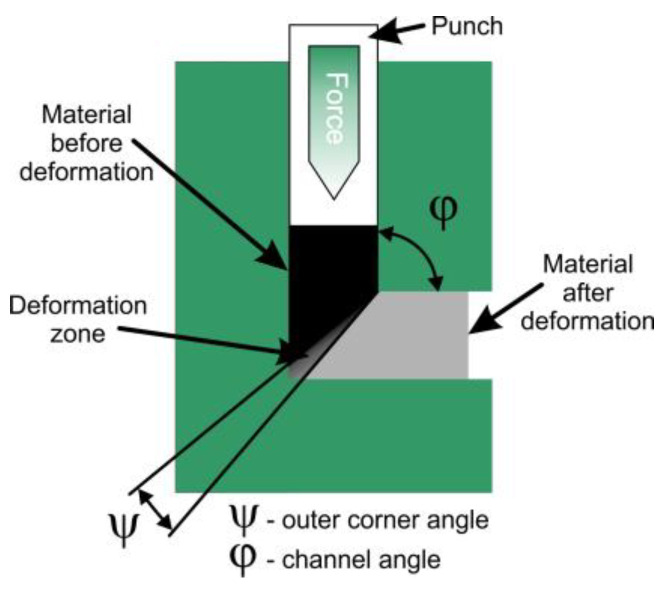
Schematic ECAP process.

Equal channel angular pressing is one of the discontinuous processes of severe plastic deformation (Figure 17) [89,90] *φ* is the angle between two equal channels, and *Ψ* is the corner angle or angle of curvature. The geometry of this tool indicates that the material is deformed by simple shear under ideal, frictionless conditions. The cross-section of the sample remains approximately equal before and after the processing step; this makes it possible to subject one sample to ECAP several times in order to achieve the highest degrees of plastic deformation. The ECAP process is an efficient method to produce bulk ultra-fine-grained or nanostructured Ti-type alloys with good mechanical properties and excellent biocompatibility [98].

It has been shown that SPD methods can be used to produce bulk nanostructured biomaterials, including cp-Ti and Ti-based alloys (Table 5) [98,99,100]. For example, the microstructure of the β-type Ti15Mo alloy can be refined using the HPT method, with a grain size below 100 nm [101].

Additionally, osseointegration of Ti alloys processed in SPDs can be improved by surface modification using various methods such as mechanical polishing, electrochemical oxidation (anodizing), thermal oxidation, electrolytic oxidation of plasma, and etching [98].

Each technique has a different set of experimental conditions that help to replicate studies. In Table 6 different parameters of synthesis, characterization methods, and reproducibility checks are presented. Synthesis parameters are defined as the crucial experimental variables that influence the outcome. Characterization methods are meant as commonly used techniques to evaluate phase, morphology, structure, and composition. Reproducibility checks help ensure reliability and consistency across multiple synthesis runs.

## 4. Consolidating the Techniques of Nanoparticles

A promising group of biomaterials is metal composite materials, among which titanium-based composites dispersion-reinforced with ceramic particles are of particular interest [27,28,29,30,31,33,83,103,104,105,106,107,108,109,110].

The following processes can be used to fabricate bulk nanobiocomposite materials from nanoparticles: hot pressing, sinter forging, hot isostatic pressing, transformation-assisted consolidation, spark plasma sintering, plasma pressure compaction, severe plastic torsional straining, microwave sintering, shock wave dynamic consolidation, plasma forming, and laser techniques.

### 4.1. Hot Pressing

**Figure 18:**
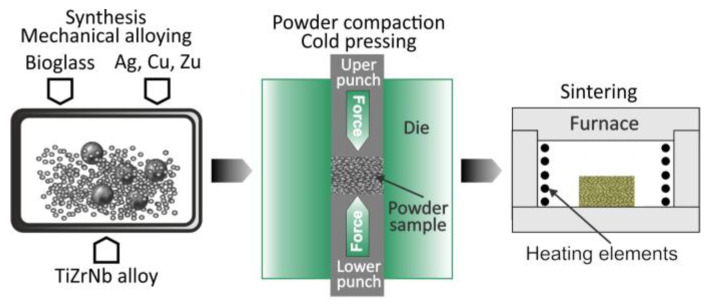
Schematic representation of the method for manufacturing Ti23Zr25Nb-9BG-Ag (Cu, Zn) biocomposite using mechanical alloying and powder metallurgy.

The hot pressing technique allows to achievement of high density with small grain sizes [109]. However, to achieve successful consolidation without grain growth, high pressures must be applied (Figure 18). The maximum size of the consolidated parts is currently limited to a cross-sectional area of 1 cm^2^, as commercial presses available to date are not capable of exceeding this limit.

### 4.2. Sinter Forging

In sinter forging, superplastic deformation and sintering occur simultaneously. Due to superplastic flow, pore closure takes place relatively rapidly. This process is limited to cylindrical shapes due to uneven density distribution across the specimen.

### 4.3. Hot Isostatic Pressing

Hot isostatic pressing (HIP) successfully consolidates coarse powder into a bulk material. The difficulty of achieving the high pressures required to drive diffusion to close pores at a faster rate than diffusion to grain growth limits the effective use of this method for nanomaterial consolidation.

### 4.4. Transformation-Assisted Consolidation

Transformation-assisted consolidation (TAC) uses pressures of several GPa. A combination of ultra-high pressure and moderate temperature with a high-volume chamber was achieved [111,112] Pressure and temperature control help to produce a sintered product with a grain size as small as the initial nanoparticles. It has been shown that bulk TiO_2_ with a grain structure at the nanoscale can be produced by high-pressure/low-temperature sintering of a metastable polymorph (anatase for TiO_2_) [112].

### 4.5. Spark Plasma Sintering

**Figure 19:**
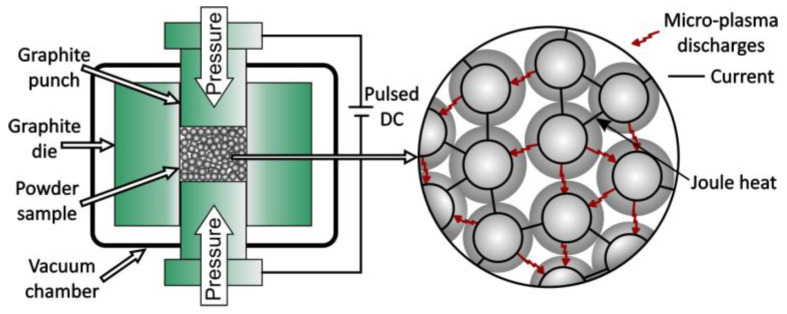
Schematic of spark plasma sintering set-up.

Spark plasma sintering (SPS) was developed for the consolidation of metallic, oxide, and ceramic powders using pulsed electric current for heating (Figure 19) [105,113,114]. Plasma is generated between intermolecular spaces that destabilize the surface oxide layers, resulting in sintering by diffusion. The SPS process was used to consolidate ultra-fine and nano-grained materials.

### 4.6. Plasma Pressure Compaction

Plasma pressure compaction (PPC) is another rapid consolidation technique used for the consolidation of nanocrystalline powders [115]. A graphite stamp can be used in this process. At the beginning of the process, a pulsed DC voltage is applied through powder compacts to create an intermolecular plasma that activates the particle surface by removing oxide layers and impurities.

### 4.7. Severe Plastic Torsional Straining

Another process for the consolidation of metal and ceramic nanoparticles is strong plastic torsional strain (SPTS) [116]. At room temperature, large plastic and shear deformations occurred under high pressure. Consolidation of SPTS powders is an efficient method for the production of metal-ceramic nanocomposites with preserved grain sizes in the range of 10–40 nm.

### 4.8. Microwave Sintering

Microwave sintering of powdered materials uses electromagnetic radiation with wavelengths from 1 mm to 1 m in free space with frequencies from 300 GHz to 300 MHz, respectively [117]. Due to faster heating in the microwave process, sintering minimizes grain growth during nanoparticle consolidation. Powders of almost all metals and alloys can be processed.

### 4.9. Dynamic Consolidation Using Shock Waves

Consolidation of metal nanopowders using shock waves from an explosive collision or high-velocity collision is a new method for processing bulk nanocrystalline materials [118,119,120]. Due to the collapse of powder agglomerates during the passage of shock waves, the particle is apparent deformation and densification. Using this method, many nanoparticles were consolidated. For example, a nanostructured bulk metal was obtained from Ti powders with a size of 40–50 nm [120].

### 4.10. Plasma Forming

The plasma spraying technique was previously used to apply protective coatings. Recently, the formation of a near-mesh shape using the plasma spraying technique was established [121,122]. Plasma spraying produces an arc that ionizes a stream of gas, creating a plasma with temperatures above 10,000 °C. Plasma forming involves melting the powder simultaneously and accelerating the molten particles to settle on a rotating mandrel. This technology enables the synthesis of a wide range of alloys, ceramics and composites [123].

### 4.11. Laser-Based Techniques

Laser energy is used to process biomaterials in a variety of forms, including laser alloying, laser surface engineering, laser sintering, and the fabrication of free-form solids [124,125,126,127]. Laser 3D printing has been applied to polymers, ceramics, and metals. Additionally, materials used for medical applications can be modified and functionalized using the direct laser interference patterning method [128]. In this way, lines, pillars, and lamellas can be produced, e.g., on titanium and polystyrene, with surface element sizes down to the sub-micrometer range.

## 5. Scaffolds

Scaffolds are one of the most important materials in tissue engineering [129,130,131,132,133]. These structures serve as a three-dimensional template for the initial attachment of cells and the subsequent formation of tissues. For medical implants, pores lead to a reduction in Young’s modulus, reduced density, and reduced osseointegration time. The main disadvantage is the decrease in mechanical strength as porosity increases.

Various manufacturing methods are used to manufacture scaffolds: binder spraying technique, direct cell recording, electrospinning, fused deposition modeling, gas foaming, inkjet printing, laser-assisted bioprinting, metal-based additive manufacturing, powder foaming, selective laser sintering, sol–gel, solvent casting/particle leaching, and stereolithography [129,130,131]. These techniques use materials such as ceramics, plastics, metals, liquids, powders, and even living cells. The scaffold provides cells with the necessary support to attach, grow, proliferate, and maintain their diverse function [132]. Examples of the techniques are given below.

In the binder spraying method, powder particles are selectively bonded and then sintered [134]. This process involves a number of complex procedures for manufacturing 3D parts, including printing the green part, removing the powder, debinding and sintering, infiltration, and finishing. Recently, cp-Ti has been used to investigate the effect of powder size distribution on the properties of green and sintered samples [135].

Direct cell writing is a laser-induced optical force that can be used to deposit particles with diameters from 100 nm to 10 μm on solid surfaces [136]. Potential applications include three-dimensional cell modelling in tissue engineering and the fabrication of biochip arrays. Recently, a distance-controlled ink (DC-DIW) approach has been established. This method integrates the method of constant interlayer distance control with traditional DIW to construct macro-scale 3D architectures from suspension ink consisting of titanium alloy powders and sacrificial additives embedded in a polymer matrix [137].

Electrospinning is one of the most popular methods for producing 1D TiO_2_ nanofibers (NFs) with high porosity, designed structures, and interesting properties. Currently, TiO_2_ NFs with porous, hollow, hierarchical, aligned structures, and self-assembled 3D nanostructures are synthesized [133,138,139].

Fused deposition modeling is a rapid prototyping method used in the production of 3D scaffolds [140,141]. The mentioned process uses a layer-by-layer deposition technique in which molten polymers or ceramics are extruded through a nozzle with a small hole that fuses with the material on the previous layer. The addition of fillers such as carbon materials, metals, ceramics, glass, and other fibrous fillers is used to strengthen the structure and improve mechanical properties.

Recently, the new rapid hot gas forming (RHGF) process of titanium alloys has been established [142]. The maximum forming temperature and pressure were over 900 °C, and 70 MPa, respectively.

Selected body parts can be printed using metal-based additive manufacturing [143]. This process, known as 3D printing, is among the most advanced manufacturing options to create complicated shapes of implants.

The production of metallic foams is of interest to the biomedical industry [144]. One of the approaches to their production is based on the use of metals/alloys in the form of micro-, nano-, or amorphous powders [145,146,147,148,149,150,151]. This process is referred to as the powder metallurgy pathway. In particular, titanium-based foam is of great interest from the point of view of supporting bone ingrowth and inducing prosthesis stabilization [145,146,147].

Selective laser melting (SLM) is a process involving the sintering of thin layers of powdered materials spread over the printing table platform using a laser [152,153]. The parameters of the laser process play an important role in the microstructure and mechanical properties of titanium samples produced by this method. Analysis of the surface structure and porosity of Ti6Al4V samples produced by SLM at different laser scanning speeds and powder layer thicknesses showed that the unstable flow of the alloy is responsible for the increased porosity and surface roughness [154].

Stereolithography is an additive manufacturing process that allows parts to be manufactured from a computer-aided design (CAD) file [155]. The designed geometry of the outer and inner pores of the structure can be built. For example, with the introduction of hydroxyapatite composites, peptide-grafted structures, cell-containing hydrogels, and modified natural polymers have been developed.

Scaffolds with controlled, interconnected porous networks and microporosity can be fabricated using the sacrificial template method (using polymers such as PMMA) and 3D printing [156,157]. The 3D printing method can be used to obtain scaffolds with a porosity of up to 90%. This technique facilitates the production of porous metals, including titanium, titanium-nickel, and tantalum.

Another method is direct ink writing (DIW) using a Delta WASP 2040 clay 3D printer [158]. For example, Ti6Al4V porous scaffolds with two scales of interconnected porosity networks were developed using the DIW process. The inks were produced by dissolving a copolymer in water to obtain a hydrogel, which was then loaded with Ti6Al4V particles [159].

New techniques for the fabrication of biomimetic porous titanium scaffolds for bone substitution have been established [160,161,162,163]. A pore size distribution in the range of 200 to 500 μm is essential and can be obtained by the space holder sintering method [162]. Deposition on materials with complex shapes, such as porous implants, can also be achieved by a biomimetic process. The process was based on heterogeneous nucleation of calcium phosphate (Ca-P) from simulated body fluid (SBF), in which titanium implants were immersed directly in the SBF solution, and a Ca-P layer was coated on the surface. Another method to improve bioactive properties is to coat the surface of Ti implants with hydroxyapatite [163]. HA deposition can be achieved by several methods, including sol–gel, plasma spraying, electrophoretic deposition, and pulsed laser deposition.

Zhang and Zou used the fiber sintering process to produce porous titanium with a three-dimensional (3D) structure, a pore size of 150–600 μm, and a porosity of 67% [162]. To increase the bioactivity of the surface, silicon-substituted hydroxyapatite (Si-HA) was coated on the surface using a biomimetic technique. Both HA-coated and silicate-coated Si-HA-titanium exhibited a significantly higher rate of bone ingrowth (BIR) than uncoated titanium.

## 6. Surface Modification Methods for Titanium

Various methods for modifying the surface of titanium are available, including electrochemical treatment and thermochemical surface treatment such as boronization, carburizing, oxidation, and nitriding [164,165,166]. Plasma modification techniques, such as physical vapor deposition and chemical vapor deposition, are also commonly applied to functionalize biomaterials [167,168]. Other energy beam surface modification techniques, such as electron beam alloying or laser and plasma techniques, are also available [49,168].

**Figure 20:**
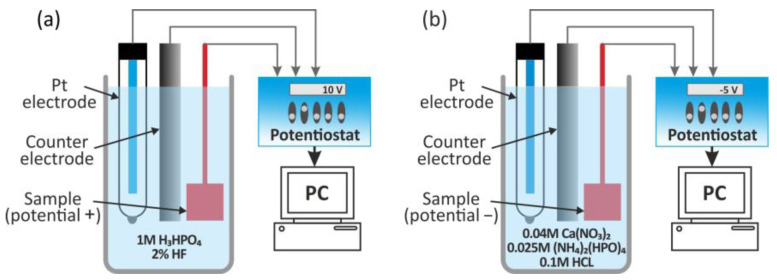
Surface modification of Ti_23_Mo-x wt %. % 45S5 Bioglass nanocomposite: (**a**) electrochemical etching at 10 V vs. OCP (open-circuit potential) for 60 min in 1 M H_3_PO_4_ + 2% HF, (**b**) cathodic deposition at −5 V vs. OCP in the electrolyte containing 0.042 M Ca(NO_3_)_2_ + 0.025 (NH_4_)_2_HPO_4_ + 0.1 M HCl.

Modification at the nanoscale can change the chemical composition and topography of the implant surface [103,166,169]. Methods for surface modification of titanium and titanium alloys include the above-mentioned processes used for morphological modifications (roughness) and for obtaining various coatings on the surface of implants (hydroxyapatite (Figure 20), biomimetic calcium phosphate, and biomolecule functionalized coatings), as well as a mixture of coatings for a combined synergistic effect.

The methods used to create nanofeatures on titanium implant materials are summarized in Table 7.

Several of these methods have already been used to modify implants available on the market [170,171]. Nanoscale surfaces have high surface energy, leading to increased initial protein adsorption, which is crucial for regulating cellular interactions on the implant surface. Surface properties also affect adhesion, along with charge distribution and material chemistry [164]. Recently, it has been observed that the roughness of titanium nanostructures itself affects the adhesion of osteoblast cells, as well as their spread and proliferation [170].

To date, a few nanoscale surface topography modifications have been used to enhance bone responses at clinical dental implants [102,172,173,174,175,176]. For example, the OsseoSpeed surface (Astra Tech AB, Molndal, Sweden) has nanostructural features resulting from the abrasive blasting of TiO_2_ followed by the patented hydrofluoric acid treatment [177]. Astra Tech TiOblast is the precursor to the OsseoSpeed surface. This surface was sandblasted with titanium dioxide particles to obtain an isotropic, moderately rough surface. OsseoSpeed obtains its additional surface features through fluoridation and slight topographic modification of the TiOblast surface [178].

Another nanoscale surface implant currently available on the clinical market involves a modification of the CaP nanoparticles of a minimally rough titanium alloy implant [177].

### 6.1. Ion Beam-Assisted Deposition

Recently, an ion beam-assisted deposition (IBAD) process that provides increased integration with the implant surface, known as high-energy sputtering, has been used to create a commercially available dental implant surface [173]. In the NanoTite process, a high-energy ion beam source directs the ion beam to the surface of the target treated with HA.

### 6.2. Microplasma Surface Alloying

To improve the surface properties of titanium and expand its clinical applications, surface alloying of boride microplasma was used to modify the surface of titanium [49,168]. For more information, see Section 3.1.

### 6.3. Anodization Process

Recently, much attention has been paid to the preparation of TiO_2_ nanotubes on titanium implants using anodization [179,180,181,182,183,184]. Titanium foil anodizing is the most advantageous method for synthesizing self-ordered arrays of TiO_2_ nanotubes. TiO_2_ nanotubes can promote the differentiation and proliferation of bone marrow mesenchymal stem cells, osteoblasts, and chondrocytes, thereby increasing the ability of the implant surface to integrate with the surrounding bone tissue.

The physical and mechanical properties of titanium dioxide nanotubes as an implantable material depend on their geometric dimensions, which can be controlled by selecting the parameters of the anodizing process, such as current density, voltage, time, electrolyte temperature, composition, and pH, and even electrolyte aging [183,184]. As shown in previous studies, the adhesion, spread, growth, and differentiation of mesenchymal stem cells are highly dependent on tube diameter [185]. A spacing of less than 30 nm with a maximum at 15 nm increases cellular activity compared to smooth TiO_2_ surfaces. Cell adhesion and proliferation were severely impaired in nanotube layers larger than 50 nm in diameter. This results in a dramatic reduction in cellular activity and a high degree of cell death.

The electrochemical and mechanical properties of hexagonal titanium dioxide (hTNT) nanotubes have recently been investigated [186]. The fabrication process involved sonoelectrochemical anodizing of titanium foil in various electrolytes to obtain titanium oxide layers with different morphology. Scanning electron microscopy revealed the formation of well-ordered hexagonal TNT with diagonals in the range of 30–95 nm and heights in the range of 3500–4000 nm. Electrochemical measurements performed in 3.5% NaCl and Ringer’s solution confirmed a more positive open-circuit potential, lower impedance, higher electrical conductivity, and a higher corrosion rate of hTNT compared to compact TiO_2_. Nanoindentation tests showed that the mechanical properties of hTNT were affected by their diagonal size, with decreasing hardness and Young’s modulus observed as the hTNT diagonal size increased, accompanied by increased plastic deformation.

Recently, bioactive anatase nanotubes of about 100 nm in size on titanium were successfully produced by anodizing and heat treatment, which improved biocompatibility, assessed on the basis of the ability to form apatite. Due to their antimicrobial properties, high biocompatibility and wettability, low roughness, excellent chemical stability, and high specific surface area, titanium dioxide (TNT) nanotubes are of particular interest [187,188].

Current fabrication approaches, such as one-step anodization, template-assisted growth, and sonoelectrochemical anodization, enable the formation of highly ordered hTNT arrays [189].

Recent results indicate that the nanoscale surface architecture of side-spacing topography can significantly affect stem cell adhesion to substrates for biomedical applications [190].

### 6.4. Bioactive Foam Scaffolds

The high stiffness of titanium, compared to bone, may promote problems with shielding against stress and lead to subsequent loosening of the implant [191,192]. Creating a hydroxyapatite coating on the titanium surface can eliminate the interface problem.

Stupp et al. produced materials known as organopathites (OAs) that contain 2–3% poly(L-lysine) to the mineral hydroxyapatite [192,193,194,195]. The addition of these macromolecules to the mineral phase mimics some natural biogenic minerals, which contain small amounts of occluded proteins that regulate crystal formation as well as harden brittle matrices.

A new method of obtaining OA on the surfaces of titanium-based implants was proposed by Spoerke et al. [196]. The resulting material was three-dimensional, porous titanium foam with excellent matrix integrity and strength. It has been shown that the deposition of organoapatite on titanium surfaces increases their colonization by bone cells.

Independently, Hirota et al. investigated the bone regeneration properties of titanium fiber mesh as a tissue-engineered material [197]. Titanium fiber mesh exhibits sufficient biocompatibility and strength [198]. In vivo experiments have shown that a thin film of HA increases osteoblast activity and bone regeneration in a titanium fiber mesh scaffold. Today, HA coatings are widely used on titanium biomaterials to improve biocompatibility and promote bone formation.

Emerging evidence suggests that combining methods can yield benefits greater than either technique alone, particularly in terms of osseointegration, mechanical bonding, bioactivity, and antibacterial properties. Although not directly paired with plasma spraying, anodization combined with other methods demonstrates clear synergistic outcomes. For instance, anodization followed by hydrothermal deposition of hydroxyapatite (HA) can mimic traditional plasma spray approaches while offering finer control. Studies have shown improved bone-like mineral deposition and integration on titanium surfaces [199].

Anodization, applied after ultrasonic acid etching, resulted in the formation of nanotube arrays on micro-roughened Ti6Al4V surfaces. This enhanced osteoblast proliferation and bone formation, surpassing the effects of either treatment alone [200].

While direct combinations of anodization and plasma spraying are less frequently documented, plasma spraying combined with other preparatory steps demonstrates tangible benefits. For instance, applying Ti or Zr interlayers before HA plasma spraying enhanced coating adhesion and corrosion resistance in 316L stainless steel implants [201].

Micro-arc oxidation (MAO)—a plasma-assisted form of anodization—is a particularly relevant approach. It combines anodic oxidation and plasma electrolytic discharge to create in situ, strongly bonded porous oxide coatings. These coatings often incorporate bioactive elements, such as calcium or phosphorus, which enhance both mechanical and biological properties. By effectively merging principles of both anodization and plasma treatment, MAO offers a single, powerful approach [202].

Combining surface modification techniques can enhance performance in various ways, such as improving mechanical bonding, enhancing osseointegration, and imparting antibacterial properties. While the exact synergy between traditional anodization and plasma spraying, a two-step sequential treatment, is less documented, analogous strategies like MAO or plasma spraying demonstrate the same principle: layered or combined methods often outperform single treatments.

Table 8 summarizes various synthesis methods used to fabricate titanium-based biomaterials and nanocomposites, highlighting their corresponding material compositions and enhanced properties. These techniques range from traditional powder metallurgy and sol–gel methods to advanced approaches like friction-stir processing, electrochemical treatments, and additive manufacturing. Each method offers unique advantages in tailoring the mechanical, chemical, and biological properties of the resulting materials, with a strong focus on improving biocompatibility, corrosion resistance, antibacterial activity, and structural performance for biomedical applications.

## 7. Conclusions and Perspectives

Nanotechnology is fundamentally changing medicine. Improvement of the physicochemical and mechanical properties of titanium-based implant materials can be achieved by controlling their microstructure. This can be carried out using top-down approaches, such as severe plastic deformation and mechanical alloy techniques. Among the improvements in mechanical properties based on the Hall–Petch relationship, the biocompatibility of materials produced in this way can also be improved. Nanogranular materials have high surface energy due to a very high number of atoms on the surface. This property may explain their completely different behavior compared to micron-sized grains. For example, osteoblasts adhere to surfaces with a roughness in the nanometer range. However, it is not only roughness but also composition and surface energy that affect the initial contact and spread of cells. Nanoscale surfaces with high surface energy lead to increased initial protein adsorption, which is very important in the regulation of cellular interactions on the implant surface. Surface properties also affect adhesion, as well as charge distribution and the chemical composition of the material. Recently, it has been observed that the roughness of the titanium nanostructure itself affects osteoblast cell adhesion, as well as their spread and proliferation. To date, several nanoscale modifications of surface topography have been used to improve bone response to clinical implants.

Current research focuses not only on changes in the composition and microstructure of the alloy but also on surface treatment. Methods for surface modification of titanium and titanium alloys include physical, chemical, and mechanical methods used for morphological modifications (roughness) and for obtaining various coatings on the surface of implants (hydroxyapatite, biomimetic calcium phosphate, biomolecule functionalized coatings), as well as a mixture of morphological changes and coatings for a combined synergistic effect.

The final goal is to improve the wear and corrosion resistance of the mentioned alloys, as well as their biocompatibility. In the case of titanium, attention is paid to enhancing the biocompatibility of commercial purity grades to avoid potential bio-toxicity of alloying elements.

Another method to alter the properties of Ti and Ti-based alloys is to produce a composite that combines the favorable mechanical properties of titanium with the excellent biocompatibility and bioactivity of ceramics. By focusing on the synthesis of nanoscale titanium-based biocomposites, many researchers have achieved nanomaterials with better mechanical and corrosion properties than microcrystalline titanium.

Electrochemical treatment provides good corrosion resistance and biocompatibility, as well as good osseointegration. The rough surface of the implant affects wettability and stimulates cell attachment and proliferation.

Currently, there is still limited evidence for the long-term benefits of nanofeatures, as promising results obtained in in vitro and animal studies have not yet been confirmed in humans. The development of nanotechnology presents numerous challenges and risks in the field of health and the environment. For this reason, a deeper understanding of science is necessary, including risk–benefit analysis and ethical considerations during the development process. However, the environmental impact of nanoparticles and their effects, including toxicity, persistence, and bioaccumulation, needs to be investigated.

The ideal implant will be based on a surface with controlled topography and chemical composition. Shortening the osseointegration time in cases of immediate strain will enable patients to undergo shorter and safer rehabilitation treatment.

It is crucial to develop titanium alloys for implants that have reduced susceptibility to bacterial colonization and do not have pathogenic effects. It appears that nanobiocomposites can be tailored to produce structures that facilitate the continuous adaptation of the host organism to the implant.

## Figures and Tables

**Table 2:** Biomaterials in recent medical applications [20,21,22,23,24].

Material Composite	Mechanical Strength	Biocompatibility	Cost & Economic Note
Ti-based nanocomposites	Very high strength, fatigue resistance	Excellent osseointegration, inert surface	High upfront cost; proven long-term value
Mg-based materials	Lower strength than Ti, close to bone modulus	Biodegradable, promotes healing, needs corrosion control	Base material cheap, implants pricier—but lifecycle savings
Mg–Ti alloys/hybrids	Improved strength & stability	Better corrosion control, biocompatible	Emerging; cost not well-established
Polymers/hybrids	Lower strength; better for non-load-bearing or soft tissue	Biocompatible; customizable	Usually low material cost; manufacturing affects price

**Table 3:** A few methods for producing nanomaterials.

Method	Approach	Key Materials	Applications
Mechanical milling	Top-down	Metals, ceramics	Nanopowders
Sol–gel	Bottom-up	Silica, bioactive glass	Bone regeneration
Self-assembly	Bottom-up	Lipids, DNA	Drug delivery, biosensors
Microbial synthesis	Biological	Ag, Au, ZnO	Antimicrobial agents
Plant extract synthesis	Biological	Metal oxide nanoparticles	Drug delivery, biosensing
Electrospinning	Hybrid	Biopolymer nanofibers	Tissue scaffolds

**Table 4:** Biogenic synthesis methods for nanoparticles.

Method	Biological Agent	Reducing/Stabilizing Components	Examples of Nano Particles	Advantages	Challenges
Plant-mediated	Plant extracts (leaves, roots, etc.)	Flavonoids, phenols, alkaloids, terpenoids	Ag, Au, ZnO, Fe_3_O_4_	Fast, scalable, eco-friendly	Variation in extract composition
Bacterial synthesis	Bacillus, Pseudomonas, etc.	Enzymes (e.g., nitrate reductase)	Ag, Au, Pd	Easy to culture, extracellular synthesis	Requires sterile conditions, slow growth
Fungal synthesis	Fusarium, Aspergillus, etc.	Extracellular enzymes, proteins	Ag, Au, TiO_2_	High yield, good scalability	Post-synthesis purification needed
Algae-mediated	Chlorella, Spirulina, etc.	Proteins, pigments, polysaccharides	Ag, Au, ZnO	Abundant biomass, rapid growth	Less studied, consistency issues
Enzyme-mediated	Isolated enzymes (e.g., laccase)	Specific enzyme action	Ag, Au	High specificity, clean synthesis	Expensive, enzyme instability

**Table 5:** Mechanical properties of conventionally processed and nanostructured cp Grade 4 Ti [102].

Processing and Treatment Conditions	Ultimate Tensile Strength [MPa]	Yield Strength [MPa]	Elongation [%]	Reduction Area [%]	Fatigue Strength at 10^6^ Cycles
Conventional Ti (as received)	700	530	25	52	340
nTi ECAP + TMT	1240	1200	12	42	620
Ti-6Al-4V ELI annealed	940	840	16	45	530

**Table 6:** Experimental conditions of the presented techniques.

Technique	Synthesis Parameters	Characterization Methods	Reproducibility Checks
Ion Beam Techniques	Ion energy, dose, beam current, target material, vacuum level, temperature	TEM, RBS, SIMS, XPS, AFM	Beam current stability, dose calibration, repeated implantations
Plasma Synthesis	Gas type, pressure, power, precursor type, substrate temp, RF/microwave frequency	SEM, TEM, XRD, XPS, OES (plasma)	Plasma density monitoring, repeated depositions, gas flow reproducibility
Pulsed Laser Deposition (PLD)	Laser fluence, pulse rate, target-substrate distance, substrate temp, ambient gas (O_2_, Ar) pressure	XRD, AFM, SEM, TEM, EDX, Raman, Ellipsometry	Laser stability, uniform target erosion, substrate rotation
Physical Vapor Deposition (PVD)	Source material, vacuum pressure, deposition rate, substrate temp, target-substrate distance	XRD, SEM, AFM, Profilometry, XPS	Thickness monitoring, rate reproducibility, substrate prep standardization
Sol–Gel	Precursor type, pH, solvent, water/alkoxide ratio, aging time, drying/calcination temp	FTIR, TGA, XRD, SEM, TEM, BET surface area	Reagent concentration control, pH monitoring, drying uniformity
Chemical Vapor Deposition (CVD)	Precursor gas, carrier gas flow, pressure, temp, reactor design, substrate	XRD, SEM, TEM, Raman, AFM, EDX	Flow control calibration, temp uniformity, gas purity checks
Hydrothermal Route	Temp, pressure, pH, precursor concentration, time, autoclave material	XRD, SEM, TEM, FTIR, Raman, BET	Sealing integrity, batch homogeneity, repeated runs
Epitaxial Growth (e.g., MBE, CVD-epitaxy)	Substrate orientation, temp, flux/gas ratio, vacuum quality, growth rate	HRXRD, TEM, RHEED, AFM, LEED	Flux monitoring, substrate prep protocols, real-time growth monitoring
Polymer Route (e.g., polymer-assisted synthesis)	Polymer type/concentration, precursor-polymer ratio, heating/calcination temp	FTIR, TGA, SEM, XRD, DLS	Consistent polymer chain length, drying conditions
Colloidal Dispersion	Surfactant, pH, ionic strength, solvent, particle concentration	DLS, Zeta potential, TEM, UV-Vis, SAXS	pH buffer consistency, mixing time, filtration steps
Microemulsions	Surfactant/co-surfactant ratio, oil/water phase ratio, temp, mixing time	DLS, TEM, SAXS, UV-Vis	Droplet size stability, consistent emulsification protocol
Solvothermal Decomposition	Solvent, precursor, temp, pressure, duration, sealed reactor type	XRD, SEM, TEM, FTIR, TGA	Pressure/temp control, precursor purity, sealing reliability
High-Energy Ball Milling	Milling time, ball-to-powder ratio, speed (rpm), atmosphere (inert or air), vial material	XRD, SEM, TEM, BET, Particle size analysis	Milling cycle repeatability, vial/ball wear checks
Mechanical Alloying	Same as high-energy ball milling, with emphasis on repeated welding and fracturing	XRD (phase), SEM (morphology), Microhardness, EDX	Homogeneity sampling, repeat milling trials
Mechanochemical Synthesis	Grinding time, force (manual or automated), stoichiometry, moisture level	XRD, FTIR, SEM, Raman	Consistent grinding duration, repeat syntheses, moisture control
Equal Channel Angular Pressing (ECAP)	Die angle, number of passes, pressing temp, strain rate, sample size	TEM, EBSD, XRD, Microhardness, DSC	Process cycle control, die wear checks, orientation control

**Table 7:** A review of the methods used to create nanofeatures on titanium implant materials.

Method	Modified Layer	Objective
Physical		
Plasma spray	Coatings such as HA, Bioglass, and boron-surface-modified layer	Improve wear-resistance, corrosion-resistance, and biological properties
Physical vapor deposition	TiN, TiC, TiCN, and thin film HA coating	Improve wear-resistance, corrosion-resistance, and biological properties
Ion implantation and deposition	Surface modification, layer, and thin film	Improve wear-resistance, corrosion-resistance, and bio-logical properties
Chemical		
Sol–gel	Thin films of calcium, phosphate, TiO_2_, and silica.	Improve wear-resistance, corrosion-resistance, and biological properties
Chemical vapor deposition	TiN, TiC, TiCN, and thin film, HAp coating.	Improve wear-resistance, corrosion-resistance, and biological properties
Anodic oxidation	TiO_2_ layers with specific surface topographies	Improve wear-resistance, corrosion resistance, and biological properties
Cathodic deposition	Ca-P deposition	Improve corrosion-resistance and biological properties
Chemical treatments such as acidic, alkaline and with hydrogen peroxide	Oxide layers, sodium, titanate gel	Remove oxides and contaminations, improve biocompatibility
Biochemical methods	Coating deposition	Induce specific cell and tissue
Mechanical		
Severe plastic deformation	Bulk ultrafine grain metals/alloys	Improve mechanical and biological properties
Mechanical alloying and powder metallurgy	Alloys with nanostructure and nanobiocomposites	Improve mechanical properties, corrosion-resistance, and biological properties

**Table 8:** Ti-based nanocomposites in recent biomedical applications [27,29,30,90,98,99,100,108,146,186,187,188,203,204,205,206,207,208,209,210,211,212,213,214,215,216,217,218,219,220,221,222,223,224,225].

Synthesis Method	Biomaterial	Properties
sol–gel method	titanium-based organic–inorganic nanocomposites	good thermal and chemical stability
selective laser melting technology	titanium-based nanocomposites doped with 0.5 wt% graphene oxide and 7.0% nanomolybdenum	enhancing the hardness and tensile properties
organic–inorganic hybrid polymerization process	titanium oxide/carbon-based nanocomposite	intermediary properties of its organic and inorganic components
solution blending method	natural rubber and TiO_2_ NPs TiO_2_/PA6 NCs polyurethane/TiO_2_ composite TiO_2_ NPs with polyethylene	excellent 100% antibacterial activity against the treatment of *E. coli*
powder metallurgy technique	graphene nanoflakes as reinforcement for the preparation of Ti6Al4V MMCs graphene as a reinforcement to produce Ti MMCs hydroxyapatite with titanium NPs	enhancement of mechanical properties enhancement of mechanical properties enhancement of mechanical properties and stability when exposed to a physiological electrolyte
photopolymerization process	chitosan-based binders modified with titanium and titanium dioxide	good buffering properties in simulated body fluids, positive effect on the mechanical properties of the binder, increase in the hydrophobicity of the material
anodization, deposition, and spin-coating methods	nanotubes containing nano-Ag and bioceramics on Ti–35Nb–2Ta–3Zr	enhanced cell adhesion, proliferation, and alkaline phosphatase activity, good antibacterial activity
electrochemical methods via Growing Integration Layers	hydroxyapatite ceramic coating on titanium alloys	improved bioactivity and adhesion of the ceramic layer
sonoelectrochemical anodization and heat treatment	anatase nanotubes with a size of approximately 100 nm on the titanium	improved biocompatibility, good antibacterial properties, high biocompatibility and wettability, low roughness, excellent chemical stability, and large specific surface area
sonoelectrochemical anodization	hexagonal TNTs with diagonals in the range of 30–95 nm and heights in the range of 3500–4000 nm	lower impedance, higher electrical conductivity, decreased Young’s modulus with increasing diagonal size of the hTNTs
ECAP	cp-Ti (α + β) titanium alloys: Ti-6Al-4V and Ti-6Al-7Nb β-Ti alloys: Ti15Mo Ti–35Nb–3Zr–2Ta shape memory alloy NiTi	improvement of the strength and fatigue properties
high-pressure torsion	Ti-bovine serum albumin nanocomposite	high hardness, increased biocompatibility
friction-stir processing	Ti-HA nanocomposite	good combination of strength and ductility by refining the grain structure with a well-developed dimple-like structure on the fracture surface
MA and space-holder sintering process	Ti–10 wt.% SiO_2_ nanocomposites and their scaffolds nanostructured Ti-10 wt.% 45S5 Bioglass-Ag composite foams	more corrosion resistant than the cp-Ti, good in vitro cytocompatibility more corrosion resistant than the cp-Ti in Ringer solution, the cells well dispersed inside the pores, reduced bacteria adhesion (*S. aureus*) in comparison with cp-Ti
MA + powder metallurgy	Ti-HA nanocomposites (HA = 3, 10, 20 vol%) β-type Ti31Mo5HA-Ag or CeO_2_ biocomposites Ti-TiB MMNCs β-type Ti-Zr-Nb alloys modified with 45S5 Bioglass and silver	increased hardness and lowered Young’s modulus compared to cp-Ti, improved corrosion resistance elastic modulus lower than cp-Ti, two times higher hardness, significantly reduced number of *S. aureus* nanoscale size effect influences the mechanical properties and the cell viability and cytocompatibility lower Young’s modulus and better corrosion properties than the cp-Ti, improved biocompatibility

## Data Availability

No new data were created or analyzed in this study.

## Abbreviations

The following abbreviations are used in this manuscript:

ABR	Accumulative Roll Bonding
AES	Auger Electron Spectroscopy
AFM	Atomic Force Microscopy
APS	X-Ray Photoelectron Spectroscopy
BET	Brunauer–Emmett–Teller Method
BIR	Bone Ingrowth Rate
BPR	Ball to Powder Ratio
Ca-P	Calcium Phosphate
CAD	Computer-Aided Design
CEC	Cyclic Extrusion Compression
CVD	Chemical Vapor Deposition
DIW	Distance-Controlled Direct Ink Writing
DC-DIW	Direct Ink Writing
DLS	Dynamic Light Scattering
DSC	Differential Scanning Calorimetry
NFs	Nanofibers
RHGF	Rapid Hot Gas Forming
SLM	Selective Laser Melting
EBSD	Electron Backscatter Diffraction
ECAP	Equal Channel Angular Processing
EDX/EDS	Energy Dispersive X-Ray Spectroscopy
ELI	Extra-Low Interstitials
FSP	Friction Stir Processing
FTIR	Fourier Transform Infrared Spectroscopy
HA	Hydroxyapatite
HEBM	High Energy Ball Milling
HIP	Hot Isostatic Pressing
HPT	High Pressure Torsion
hTNTs	hexagonal Titanium dioxide Nanotubes
IBAD	Ion Beam Assisted Deposition
LEED	Low-Energy Electron Diffraction
MA	Mechanical Alloying
MAC/F	Multi Axial Compression/Forging
MAO	Micro-Arc Oxidation
MCS	Mechano-Chemical Synthesis
MDF	Multi Directional Forging
MMNCs	Metal Matrix Nanocomposites
MWCNTs	Multi-Walled Carbon Nanotubes
NPs	Nanoparticles
OA	Organo Apatite
OES	Optical Emission Spectroscopy
PAS	Plasma Activated Sintering
PLD	Pulsed Laser Deposition
PMMA	Polymethyl Methacrylate
PPC	Plasma Pressure Compaction
PVD	Plasma/Physical Vapor Deposition
RBS	Rutherford Backscattering Spectrometry
RCS	Repeated Corrugation and Straightening
RHEED	Reflection High-Energy Electron Diffraction
SAXS	Small-Angle X-Ray Scattering
SBF	Simulated Body Fluid
SCPL	Solvent Casting Particulate Leaching
SEM	Scanning Electron Microscopy
SIMS	Secondary Ion Mass Spectrometry
SPS	Severe Plastic Deformation
SPS	Spark Plasma Sintering
SPTS	Severe Plastic Torsional Staining
TAC	Transformation Assisted Consolidation
TE	Twist Extrusion
TEM	Transmission Electron Microscopy
TGA	Thermogravimetric Analysis
TMT	Thermomechanical Processing
UFG	Ultra-Fine Grains
UV-Vis	Ultraviolet–Visible Spectroscopy
XPS	X-Ray Photoelectron Spectroscopy
XRD	X-Ray Diffraction
